# Effect of Thermal Ageing on the Impact Damage Resistance and Tolerance of Carbon-Fibre-Reinforced Epoxy Laminates

**DOI:** 10.3390/polym11010160

**Published:** 2019-01-17

**Authors:** Irene García-Moreno, Miguel Ángel Caminero, Gloria Patricia Rodríguez, Juan José López-Cela

**Affiliations:** Escuela Técnica Superior de Ingenieros Industriales, INEI, Universidad de Castilla-La Mancha, Campus Universitario s/n, 13071-Ciudad Real, Spain; irene.gmoreno@uclm.es (I.G.-M.); gloria.rodriguez@uclm.es (G.P.R.); juanjose.lopez@uclm.es (J.J.L.-C.)

**Keywords:** thermal ageing, thermoset matrix, carbon-fibre-reinforced composites, glass transition temperature, compression after impact response, ultrasonic C-scanning

## Abstract

Composite structures are particularly vulnerable to impact, which drastically reduces their residual strength, in particular, at high temperatures. The glass-transition temperature (*T*_g_) of a polymer is a critical factor that can modify the mechanical properties of the material, affecting its density, hardness and rigidity. In this work, the influence of thermal ageing on the low-velocity impact resistance and tolerance of composites is investigated by means of compression after impact (CAI) tests. Carbon-fibre-reinforced polymer (CFRP) laminates with a *T*_g_ of 195 °C were manufactured and subjected to thermal ageing treatments at 190 and 210 °C for 10 and 20 days. Drop-weight impact tests were carried out to determine the impact response of the different composite laminates. Compression after impact tests were performed in a non-standard CAI device in order to obtain the compression residual strength. Ultrasonic C-scanning of impacted samples were examined to assess the failure mechanisms of the different configurations as a function of temperature. It was observed that damage tolerance decreases as temperature increases. Nevertheless, a post-curing process was found at temperatures below the *T*_g_ that enhances the adhesion between matrix and fibres and improves the impact resistance. Finally, the results obtained demonstrate that temperature can cause significant changes to the impact behaviour of composites and must be taken to account when designing for structural applications.

## 1. Introduction

Fibre-reinforced composite materials have become relevant in aerospace, automotive, wind energy, marine, and civil engineering applications due to their high specific stiffness and strength, corrosion resistance, and fatigue performance [1,2,3,4,5,6]. One of the major factors limiting the design of structures made from current carbon fibre-epoxy systems is the susceptibility of the material to impact damage in the form of matrix cracking, multiple delaminations, and fibre breakage [7,8,9]. Fibre failure (intra-laminar failure) affects mainly tensile strength, while delamination (interlaminar damage) decreases mainly compression. When compressive stress is applied, the interaction between the delaminations and fibre damage can have a considerable detrimental effect on the performance [7,10,11,12]. Low velocity impact damage is especially dangerous because of its difficult detectability and it is potentially a source of mechanical weakness that can propagate and cause considerable overall strength reduction. Compression after impact (CAI) testing is of great interest within the aeronautical industry, since the residual compressive strength of the damaged component is the property that decreases the most [9,13,14,15,16].

Compression after impact tests consist of uniaxial compression tests made at room temperature on an impacted specimen according to the standard ASTM D7137 [17]. This CAI test methodology employs a laminate thickness greater than 4 mm. However, many laminates from the aerospace industry are thinner. The typical thickness of laminates in the horizontal tail plane, vertical tail plane, and fuselage are between 2 and 6 mm, and even zones of these primary structures may be less than 2 mm thick [18]. A non-standard CAI device based on the design of Reference [19] is used in this work to avoid geometry modification and ensure compression failure instead of global buckling.

Furthermore, many aircraft components, such as aero-engine covers or motorsport applications can reach temperatures considerably higher than room temperature during service. Moreover, other elements often work at high temperatures for long periods, being necessary to ensure the stability of the material during service. One of the most important properties in polymers is the glass-transition temperature because at this temperature the polymer undergoes a decrease in density, hardness, and rigidity, becoming more ductile and elastic. Temperature can affect the elastic properties of the epoxy resin causing a change in the energy absorption capacity, and consequently, in the failure mechanism of the composite laminate. The typical service temperature of polymeric composite materials based on thermosetting resins would preferably be lower than the glass-transition temperature (*T*_g_), but can be applied in service at temperatures above the *T*_g_ [20]. This is the case for carbon-fibre-reinforced polymer (CFRP) laminates used in aircraft and space structures, where the material can be exposed to air at −73 °C to 80 °C or the space at −140 °C to 120 °C [21]. Unlike thermoplastic polymers that can be deformed plastically as many times as necessary without disturbing the initial properties of the material, thermosetting polymers are susceptible to thermal degradation. The effect of thermal ageing on the mechanical performance of polymer composites has been studied a limited number of times [22,23,24,25,26,27,28,29,30,31,32,33,34].

Thus, the study of the impact behaviour of CFRP materials that are exposed to high temperatures is essential. Considerable research has been devoted to analysing the impact properties and post-impact compression behaviour with a view to improving impact energy tolerance [1]. In particular, the influence of the laminate thickness and ply stacking sequence on the impact tolerance of laminated composites have been studied in several works [5,19,35,36,37,38]. However, the majority of these studies are undertaken at room temperature conditions and they have not considered the effect of temperature [39]. Some authors have performed quasi-static and dynamic tests to characterize the impact behaviour of glass-epoxy tubes at different temperatures [40]. In other studies, the effect of impact energy on the magnitude of the damage of carbon-epoxy at both high and low temperatures has been evaluated [41,42]. They proved that temperature significantly influences the impact damages of CFRP laminates and observed that delamination areas and transverse cracking increased at lower temperatures and the critical delamination energy increased as the temperature increased. Hence, it is important to understand the effects of temperature on impact resistance, which has received limited attention in the literature. Moreover, the linkage between thermal ageing and damage tolerance is still not fully understood due to the complex manner in which damage propagates within the laminate.

In this study, the characterization and the assessment of the effect of thermal ageing on the damage response of CFRP laminates subjected to low velocity impact loading is evaluated. Temperatures below and above the glass-transition temperature of the epoxy resin (195 °C) [43] and different periods of time were considered for the thermal ageing treatments. Drop-weight impact tests at low velocity energy were performed to cause barely visible impact damage (BVID) and to determine the impact response of CFRP laminates. Ultrasonic C-scanning technique was used in order to identify and measure the damage area. Finally, the compressive strength after impact (CAI) for the different aged specimens was evaluated using a non-standard device.

## 2. Materials and Methods 

### 2.1. Materials and Specimen Preparation

The specimens were fabricated from commercially available carbon/epoxy pre-impregnated laminates (Hexcel Composites Ltd., Stamford, CT, USA) [44]. The pre-preg tapes were made of unidirectional (UD) continuous high tensile strength carbon fibres IMA-12K and M21E epoxy resin, provided in rolls 300-mm wide and 0.262-mm thick. This material, named as M21E/34%/UD268/IMA-12K, is a unidirectional pre-preg used in Airbus A350 XWB primary structures (wing spars and wing covers, fuselage sections, keel beam, and central wing box). It has a resin content of 34% by weight and a fibre weight of 268 g/m^2^. The M21E epoxy resin is a thermosetting polymer that shows excellent damage tolerance, especially at high energy impacts so it is suitable for aeronautical applications [3,8]. The basic in-plane stiffness and strength of the M21E/IMA unidirectional laminate under tensile and compressive loading are presented in Table 1. These properties have been obtained from previous studies [3,5].

The CFRP laminates were laid up by hand in 200-mm wide by 200-mm long plates following the quasi-isotropic stacking sequence [0/90/±45]_2s_ with fibres oriented along the principal directions. Each laminate consists of 16 plies which corresponds to a total thickness of 4 mm, each one being the thickness of 0.25 mm approximately. 

**Figure 1:**
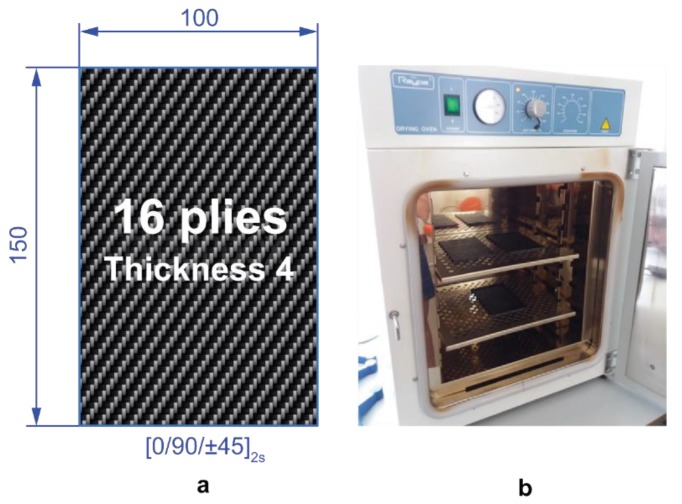
(**a**) Standard impact specimens according to ASTM D7136 recommendations. (**b**) Programmable oven used for the thermal ageing treatments.

The standard cure cycle recommended by Hexcel Composites Ltd. was used for these laminates. The plates were cured at 7 bar hot-pressing system together with slow heating (3 °C/min), hold at 180 °C for 120 min, and followed by cooling at 5 °C/min. After the post-curing, the panels were ultrasonically C-scanned to ensure panel integrity. The ASTM D7136 [17] drop weight method was followed for testing impact specimens. The characteristic impact specimen length and width were 150 mm and 100 mm, respectively (Figure 1a). Thermal ageing treatments were performed in an oven at different temperatures (Figure 1b). For that purpose, the chosen temperatures were 190 and 210 °C, below and above the glass transition temperature of the epoxy resin (195 °C), respectively. The effect of ageing was evaluated at two different periods of time: 10 days and 20 days, and the results were compared with those obtained from non-aged laminates. For each sample, three specimens were tested, and the average results were taken as the final values.

### 2.2. Low Velocity Impact Testing

**Figure 2:**
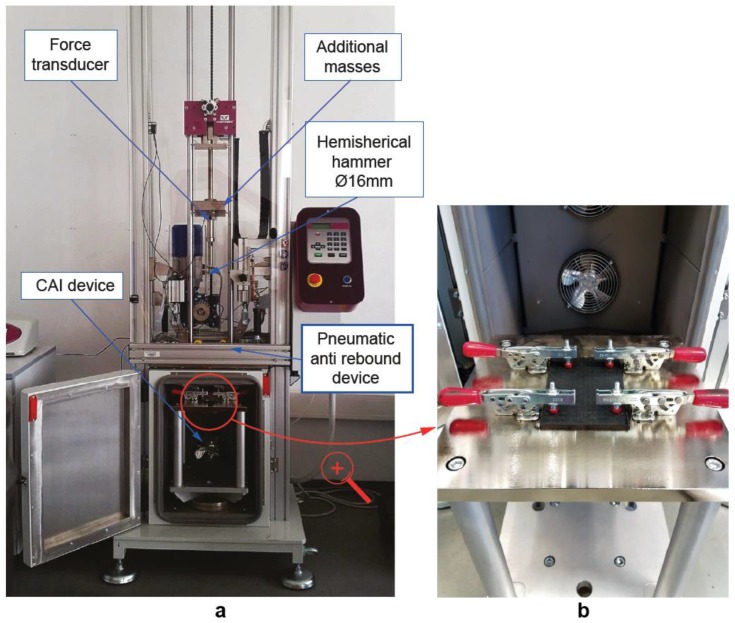
Experimental setup for drop-weight impact test. (**a**) Drop-weight column device. (**b**) Compression after impact (CAI) support fixture according to ASTM D7136 standard.

Impact tests were performed in an instrumented drop-weight tower INSTRON CEAST 9340 (Instron Ltd., Norwood, MA, USA) (Figure 2a) according to the standard. This method of damage introduction determines the damage resistance of multidirectional composite laminates subjected to a drop-weight impact event. Furthermore, it simulates typical in-service damage on composite aircraft structures, such as accidentally dropping a tool during maintenance or fabrication, bird strikes or hailstorms [45]. The indenter consisted of a hemispherical head with a 16-mm diameter and a mass of 4.5 kg. The specimens were impacted on its smoother side to facilitate the measurement of the indentation depth. In addition, each specimen received a single impact, and the rebound was prevented by an anti-rebound system. The maximum impact energy is limited by the adjustable falling weight. The specimens were clamped to the drop tower fixture using a CAI support fixture with four toggle clamps (Figure 2b). All clamps had rubber attachments to avoid damaging the specimen during clamping. The impact location was located in the centre of the specimen. The design of the testing was restricted to the analysis of low velocity below 5 m/s to avoid penetration and the energy levels were selected in order to cause BVID in non-thermal aged specimens. Some initial tests at different energies were required to select the level of energy which causes BVID. Finally, the selected impact energy was 20 J, with a drop height of 0.46 m. Impact velocity was calculated according to the previous parameters.

**Figure 3:**
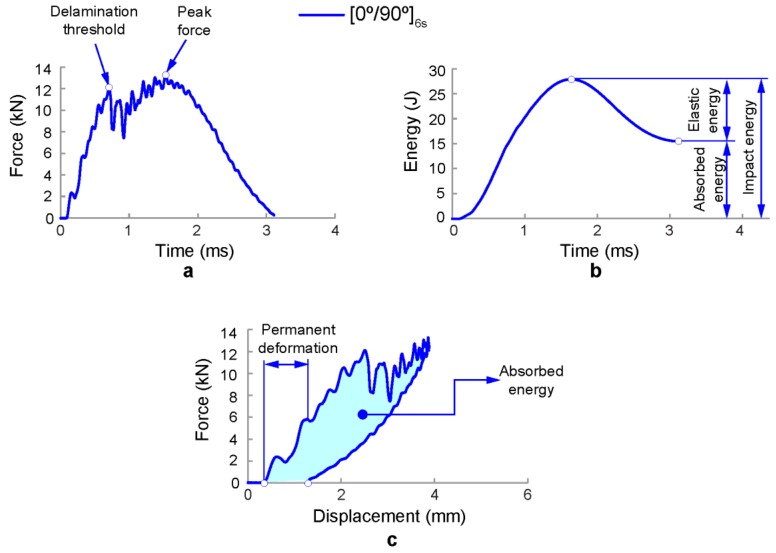
Impact damage response histories of typical composite laminates under drop-weight impact test: (**a**) force–time history, (**b**) energy–time history, and (**c**) force–displacement history [3].

Impact resistance can be determined in terms of the absorbed impact energy, peak force, peak deformation, duration of impact contact, and the resulting extent and type of damage. The damage response parameters can be obtained using the recorded impact histories of force-time, force-displacement, and absorbed energy-time (Figure 3). Peak force refers to the maximum value of force registered during the contact event between the impactor head and the specimen and the absorbed energy is the amount of energy transferred from the impactor to the specimen at the end of the test. Figure 3a depicts a typical force–time history of a drop-weight impact test. In the initial phase of contact, slight oscillations can be observed due to the elastic vibration induced by the initial contact between the impactor and the composite laminate. After that, some oscillations are observed which indicates the presence of damage. The first abrupt drop of force is due to a reduction of bending stiffness because of the brittle impact damage behaviour of CFRP and the delamination threshold is reached. After the first drop, the contact force increases again. Smaller force drops indicate crack growth in the specimen. The maximum contact force is reached at the peak force. The impactor then bounces back, and the load is reduced to zero. Hence, the impact force history of the low-velocity impact event provides important information regarding the damage initiation and propagation [12]. Figure 3b shows the absorbed energy–time relationship. The initial kinetic energy of the impactor is transferred to the composite plate once contact is made. During the impact event, part of this energy is the absorbed energy by the plate in the form of elastic deformation (elastic energy). The energy in excess is dissipated through several failure mechanisms, such as fibre breakage, delamination, fibre-matrix debonding, and matrix cracking. Hence, the absorbed energy by the specimen is an indication of the magnitude of damage [11]. Finally, force–displacement curves are shown in Figure 3c. The first part of the curve represents the stiffness of the non-damaged laminate. The second part of the curve depicts the unloading phase with elastic recovery. In addition, some permanent deformation can be observed. In this case, a part of the impact energy has contributed to form internal damage within the composite laminate.

### 2.3. Phased Array Ultrasonic Testing

**Figure 4:**
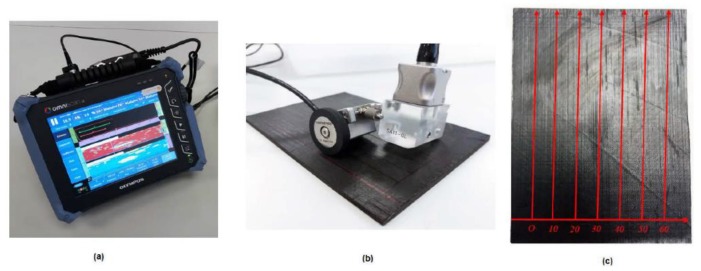
Experimental setup of phased array ultrasonic testing. (**a**) Olympus Omniscan SX. (**b**) Phased-array transducer of 64 elements at 5 MHz with coupled encoder. (**c**) Details of the raster scan scheme for the composite laminates.

Phased array ultrasonic inspections were performed using the Olympus OmniScan SX (Olympus Ltd., Tokyo, Japan) equipment with a phased-array transducer of 64 elements at 5 MHz (Figure 4a). Phased array technique presents some advantages compared to a conventional transducer, such as the reduction of the number of transducers to be used in an inspection, the ability to inspect areas with curvature and complex geometric variations, and better precision in the measurement of the defect position. Because of multi-element signal focusing, phased array ultrasonic testing shows better characteristics of attenuation and resolution of ultrasonic signals [46,47,48]. Before inspection, data acquisition requires the calibration of propagation velocity, attenuation, and acoustic impedance because these factors affect significantly the propagation of ultrasonic waves. The details of the set-up and calibration of C-scanning ultrasonic technique can be found in a previous work [49]. In the current study, the stepped block calibration technique was carried out before the C-scanning ultrasonic test to ensure an appropriate configuration to obtain accurate results. Three calibration steps were required: propagation velocity, wedge delay, and sensitivity. Calibration requires the use of a pattern with the same material of the specimen tested to reproduce the same conditions of attenuation and wave propagation. The propagation velocity (*V*) of the longitudinal waves through the material must be calibrated first and depends on the elastic modulus *E*, material density ρ, and Poisson coefficient ν according to the equation.

(1) $$V=\;\sqrt{\frac{E}{\rho }\cdot \frac{1-\nu }{\left(1+\nu \right)\cdot \left(1-2\nu \right)}}$$

The second step is the calibration of the wedge delay that sets the zero position on the face of the wedge in contact with the tested part. Finally, the sensitivity calibration provides a clearer image visualization because of signals with similar amplitude. Other factors that affect the propagation of the ultrasonic waves are the attenuation and the acoustic impedance. Attenuation is caused by the absorption, dispersion, and divergence of the beam and the acoustic impedance is related to the amount of beam that is reflected in the interface with a second medium as a discontinuity in the material.

After calibration, the instrument can recognize defects in the material comparing the height of a discontinuity echo with the reference echo. Calibration requires the use of a pattern with the same material of the specimen tested in order to reproduce the same conditions of attenuation and wave propagation.

The purpose of the ultrasonic inspection was to locate and measure the area of the laminate affected by the low-velocity impact. Internal delamination causes the beam reflection due to a discontinuity in the material. The C-Scan images provide a complete view of the damage extension after each impact test. A mechanical scanner with encoders was used to track the transducer coordinates to the desired index resolution (Figure 4b). The method selected for the inspection was a raster scan as depicted in Figure 4c. Several sweeps have been performed to obtain a complete view of the damage extension.

### 2.4. Compression after Impact (CAI) Test

**Figure 5:**
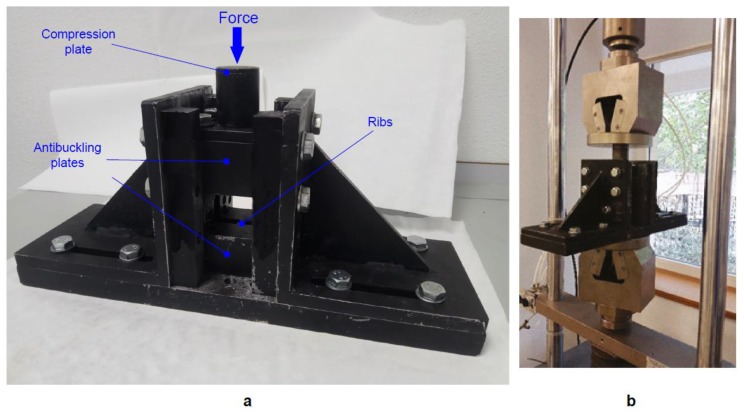
(**a**) Description of the non-standard adjustable CAI device proposed in this study. (**b**) Experimental setup for CAI testing.

Compression after impact tests were performed in order to evaluate the influence of temperature and time of thermal ageing on the residual strength of CFRP laminates. Compression after impact tests were carried out on a universal electromechanical MICROTEST EM1/100/FR testing machine with a 100 KN load cell at a constant displacement rate of 0.5 mm/min. The ASTM 7136 [17] proposes a standard CAI device for testing polymer matrix composite materials with a maximum thickness of 4 mm. However, a non-standard CAI device was designed in a previous study [19] to ensure that the failure (local buckling) does not occur in the top or bottom edges of the specimen due to compression-shear when testing specimens with a thickness lower than 4 mm. Because of its design, this CAI fixture ensures compression failure instead of global buckling and allows damage propagation perpendicular to the applied load. Figure 5 shows a schematic description of the non-standard CAI device and the experimental setup for CAI testing. The device is composed of a support structure with two pairs of anti-buckling plates, containing a set of vertical ribs that stabilize the specimen during test, increasing the buckling load. Anti-buckling plates are divided in two parts and they are fixed to the loading plates. The specimen is placed between the two pairs of anti-buckling plates, being supported by all the vertical ribs. Between the anti-buckling plates, a square central gap allows deformation during compression. The vertical ribs have sharp edges to minimize friction by reducing contact area. The compression after impact strength (σ_CAI_) was calculated based on the ultimate load (P_max_) and the specimen cross-section dimension (σ_CAI_ = P_max_/*b* × *h*), where *b* and *h* were the specimen width and thickness, respectively.

## 3. Results and Discussion

The main effects of thermal ageing on the impact damage resistance and tolerance of CFRP laminates are summarized in the following sections.

### 3.1. Effect of Ageing on the Impact Damage Resistance of CFRP Composite Laminates

**Figure 6:**
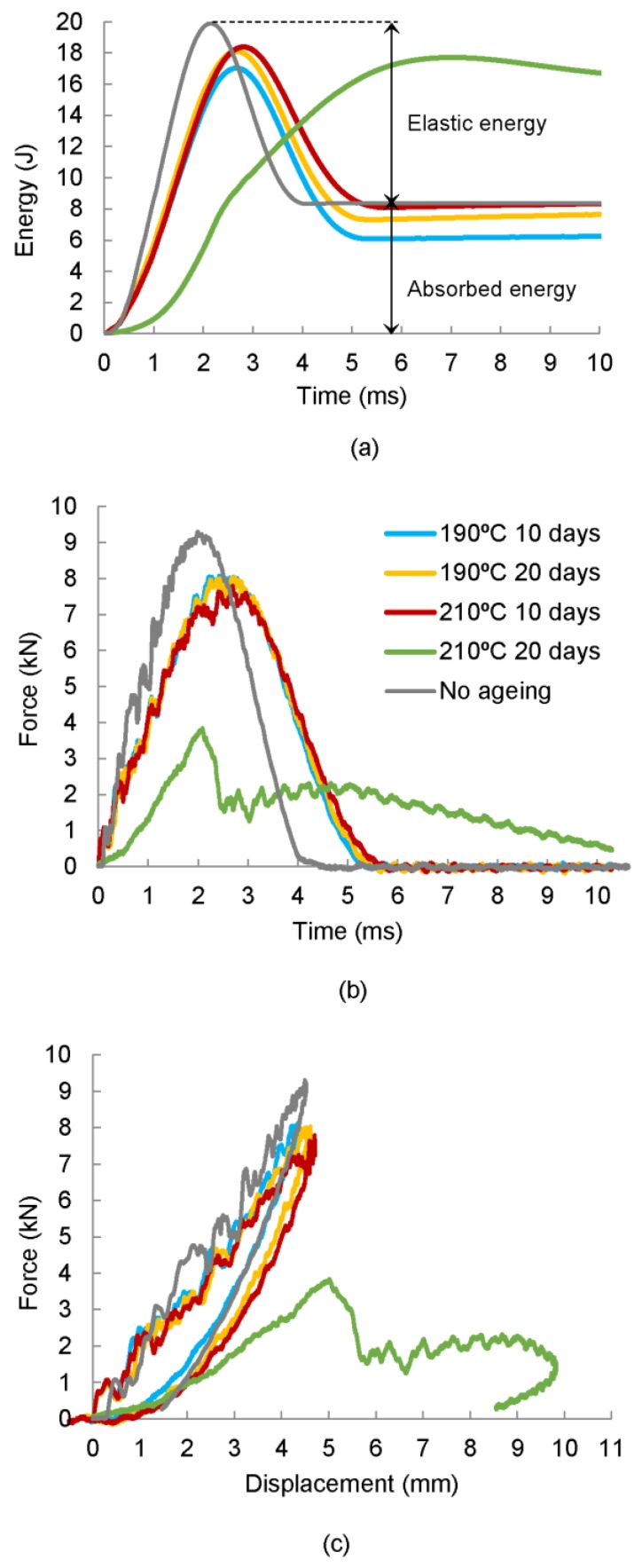
(**a**) Average energy and (**b**) force histories and (**c**) force–displacement curves of non-aged [0/90/±45]_2s_ quasi-isotropic composite laminates with different ageing processes.

**Figure 7:**
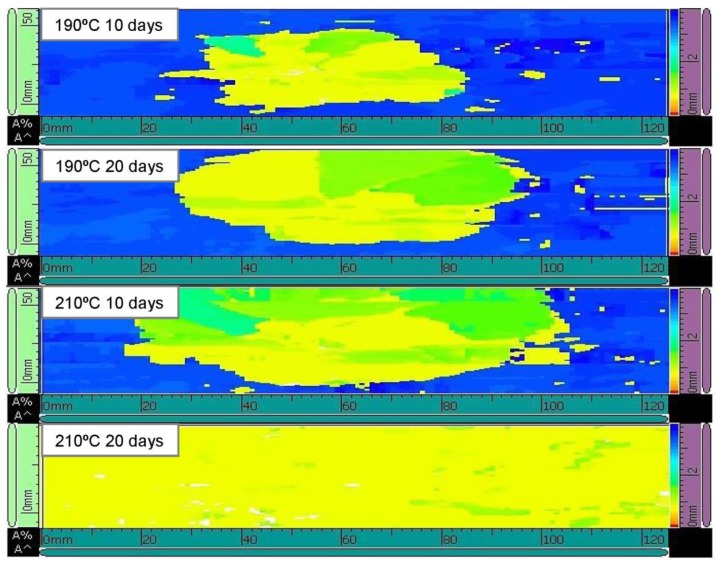
Ultrasonic C-scanning images of the damaged area of [0/90/±45]_2s_ quasi-isotropic composite laminates aged at different temperatures and periods of time. The effect of ageing on the impact damage response. (E_impact_ = 20 J).

The influence of temperature and time of ageing on the impact damage response of [0/90/±45]_2s_ quasi-isotropic CFRP laminates are analysed in this section. Two different temperatures were used in this study: T_1_ = 190 °C, which is a temperature below the transition temperature, and T_2_ = 210 °C which exceed such temperature. Temperature levels modify the elastic properties of composite laminates which affects the energy-absorbing capacity of this material. In order to compare and characterize the impact damage resistance of thermal aged quasi-isotropic CFRP laminates with the impact resistance of non-aged specimens, some representative force–time and energy–time histories, and force–displacement curves of quasi-isotropic laminates aged at different temperatures and periods of time are depicted in Figure 6. As a general remark, the impact energy is absorbed in two different ways in a composite material: as elastic deformation and as dissipated energy in the form of permanent damage. At the beginning of the test, the energy curve rises until its maximum value, which is connected to the impact energy. Once the absorbed energy–time curve reaches the maximum value, the energy decreases until it becomes stable. The value of energy at the end of the test corresponds to the permanent absorbed energy through various damage mechanisms. In addition, Figure 7 depicts ultrasonic C-scanning images corresponding to the impacted area of the CFRP laminates previously analysed. The maximum force and absorbed energy dissipated in form of permanent damage, as well as the damage extension, in form of delaminations, for the different impact specimens are tabulated in Table 2.

In this study, two types of behaviour in relation to the maximum force (peak force) and the absorbed energy in the form of damage with permanent deformation were observed in the curves due to thermal ageing. It was found that non-aged specimens absorbed more energy in the form of permanent damage than those aged at 190 °C (Figure 6a). This fact can be related to a process known as post-curing which occurs at temperatures lower than the glass transition temperature and improves the mechanical properties of the laminates. The loss of mechanical properties of the material is an indication that the first stage of consolidation has ended, and a second stage of degradation has started, in which the oxidation of the epoxy matrix and the decomposition of the fibre interface take place [50]. The curves reveal that the effect of ageing is significant for temperatures above the glass transition temperature and long periods of time (210 °C and 20 days), which is evident in the reduction of the maximum force in the force–time history (Figure 6b), as well as the loss of stiffness associated with a lower slope in the force–displacement curve (Figure 6c). These observations were in accordance with previous works [51]. A general increasing trend of the absorbed energy was observed as the severity of the ageing treatment increases, being especially remarkable the value of absorbed energy for the specimens aged at 210 °C and 20 days that have almost completely lost the ability to absorb elastic energy (Figure 6a). The results demonstrate that the impact response of composite laminates changed significantly as the temperature and time of ageing increase.

The impact energy value in this study (20 J) is not enough to cause visible damage but promotes internal damages areas in the form of delaminations. Hence, higher absorbed energy causes larger damaged areas, as C-scanning images shown in Figure 7. It should be noted that the most undesirable situation corresponds again to the specimen aged at 210 °C and 20 days because severe delaminations, which expand over the entire specimen, are observed. 

Hence, it can be affirmed that impact damage resistance of composite materials with polymeric matrix are reduced gradually for temperatures above the transition temperature as time of ageing increases because of a degradation of the matrix and the breakdown of the fibre/matrix interface. However, a consolidation phase occurs at temperatures below the glass transition temperature due to the curing reaction that facilitates the adhesion between the fibres and the matrix, maintaining or even improving the mechanical properties of the laminates. These results have confirmed the observations of previous studies [50,52,53].

### 3.2. Effect of Ageing on the Impact Damage Tolerance of CFRP Composite Laminates

**Figure 8:**
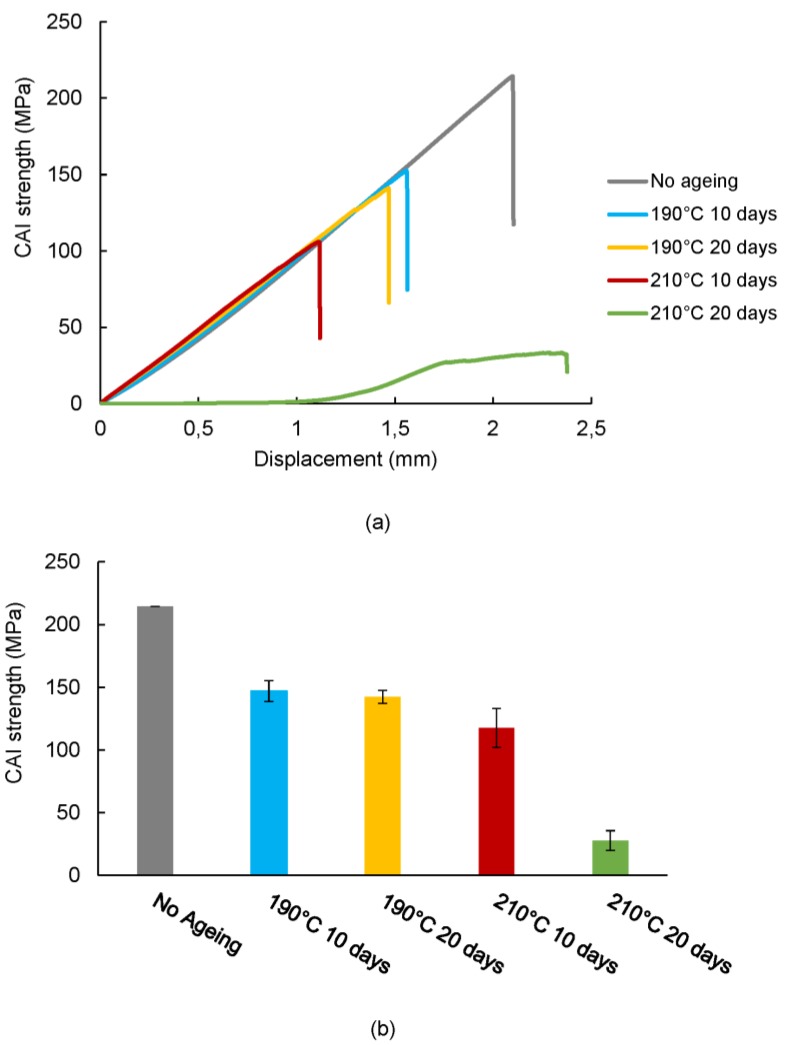
CAI test of thermal-aged [0/90/±45]_2s_ quasi-isotropic laminates. (**a**) Averaged CAI stress–displacement curves. (**b**) Average CAI results and standard deviation.

The influence of temperature and time of ageing on the impact damage tolerance and compressive failure mechanism of CAI specimens were analysed in this section. Figure 8 shows the effect of ageing on the CAI strength of quasi-isotropic laminates. As a general remark, specimens that have been exposed to ageing processes present a reduced compressive strength with respect to non-aged specimens. It was observed that both temperature and time of ageing affect gradually the residual strength. It should be notice that specimens aged for 20 days at 210 °C have been specially affected and a reduction of their residual strength from 214.47 to 27.87 MPa (87%) was observed. Nevertheless, a significant loss of compressive strength is also observed at temperatures slightly below the glass transition temperature. Despite the consolidation stage observed in the impact damage resistance of aged CFRP laminates, the CAI strength values are reduced even for ageing at temperatures below the glass transition temperature. Thermal ageing causes a progressive loss of the properties of the matrix and the degradation of the fibre/matrix interface. This effect becomes more noticeable as the ageing time and temperature increases and causes mainly a reduction of the interlaminar resistance. These results were in accordance with previous works [54,55,56]. 

**Figure 9:**
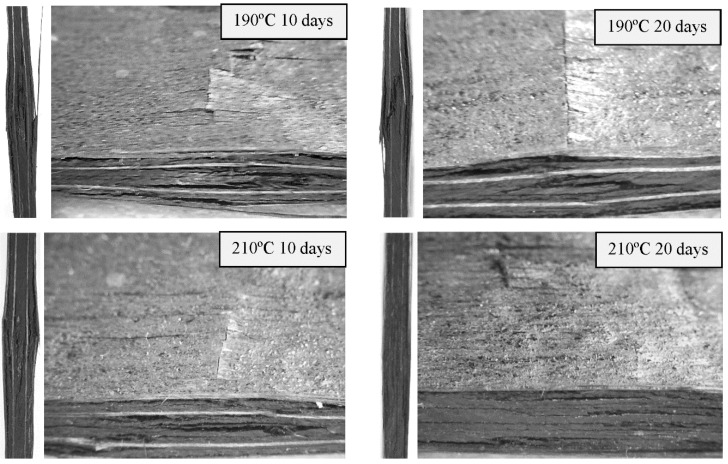
CAI damage profiles during the CAI test of [0/90/±45]_2s_ quasi-isotropic laminates aged at 190 °C and 210 °C for 10 and 20 days. An impact load of 20 J was applied on the front face.

**Figure 10:**
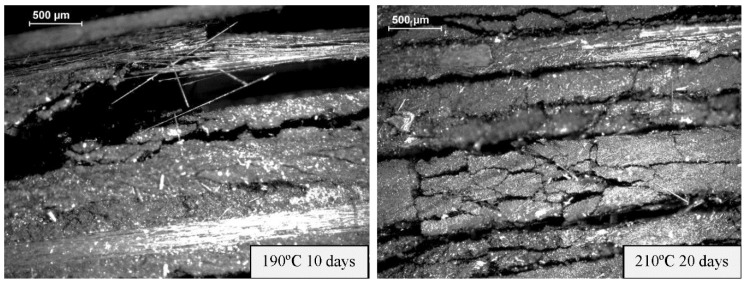
Details of the CAI damage profiles during the CAI test of [0/90/±45]_2s_ quasi-isotropic laminates aged at 190 °C for 10 days and 210 °C for 20 days.

Finally, failure mode of thermal-aged quasi-isotropic laminates [0/90/±45]_2s_ subjected to compression loading are presented in Figure 9 and Figure 10. Compression crack initiates around the impact zone located in the central section where delamination causes by the impact acts as stress concentrator, resulting in shear compression failures. Final failures for all the different aged specimens show similar behaviour (Figure 9), but impacts at the same energy cause delaminations and more extensive matrix cracking in aged specimens at 210 °C and 20 days (Figure 10), decreasing the mechanical properties of the polymer matrix composites, and in particular, impact damage tolerance. 

## 4. Conclusions

The effect of thermal ageing on the low-velocity impact damage resistance and tolerance of CFRP laminates was studied. For that purpose, several ageing treatments at temperatures both below and above the glass transition temperature (195 °C) were carried out for different periods. Drop-weight impact and CAI tests were performed in a drop weight column and a non-standard device, respectively. Significant differences were found between the non-aged specimens and the aged ones. 

The results showed that thermal ageing below the glass transition temperature for short periods of time leads to a process called post-curing which consolidates the CFRP laminate improving the adhesion between the fibres and the matrix and slightly increases the impact resistance. However, the degradation of the matrix occurs for temperatures that exceed the glass transition temperature, which implies the decomposition of the fibre matrix interface and the reduction of the impact damage resistance. 

With respect to the parameters governing the impact damage tolerance in terms of CAI strength, the effect of temperature and time of ageing cause a progressive decrease of mechanical properties, but no sign of consolidation stage were observed.

Finally, the results depicted a significant change on the impact damage resistance and impact damage tolerance of aged CFRP laminates at 210 °C for 20 days compared to the rest of the samples. It can be concluded that both temperature and time of ageing are critical parameters of design especially at temperatures above the glass transition temperature that can affect the in-service behaviour of polymeric matrix composites structures when they are exposed to high temperatures for long periods. 

## Figures and Tables

**Table 1:** Stiffness and strength properties of the M21E/IMA carbon fibre/epoxy system [3,5].

**E_11T_ (GPa)**	**E_22T_ (GPa)**	**G_12_ (GPa)**	$\sigma_{11T}\;$ **(MPa)**	$\sigma_{22T}\;$ **(MPa)**	$\tau_{12}\;$ **(MPa)**
177.5	11.8	5.2	3050.8	56.1	94.2
**E_11C_ (GPa)**	**E_22C_ (GPa)**	**ν_12_**	$\sigma_{11C}\;$ **(MPa)**	$\sigma_{22C}\;$ **(MPa)**	
146.1	7.4	0.39	1500.5	200.4	

**Table 2:** Impact results and damage morphologies of impacted CFRP specimens. Standard deviation of non-aged specimens is depicted in brackets. * The results of non-aged samples were extracted from Reference [3].

Temperature (°C)	Time (days)	F_max_ (N)	E_absorbed_ (J)	Damage extension (mm × mm)
Non-aged *	-	9212.25 (68.90)	8.23(0.30)	90.25 (0.24) × 67.84 (0.19)
190	10	7955.93	7.64	56.09 × 51.05
		7898.06	7.63	69.00 × 60.52
		8129.51	6.25	53.72 × 51.71
	20	8036.93	7.68	78.05 × 68.93
		8742.84	6.79	82.50 × 63.82
		8291.52	7.72	69.04 × 62.37
210	10	7793.91	8.38	91.49 × 52.48
		7799.70	8.39	81.88 × 67.93
		8146.87	7.86	90.82 × 64.63
	20	3847.78	16.24	Failure
		3841.99	16.83	Failure
		3552.68	16.47	Failure
